# Effects of Uric Acid on Lipid Levels in CKD Patients in a Randomized Controlled Trial

**DOI:** 10.4021/cr263w

**Published:** 2013-05-09

**Authors:** Rodney G. Bowden, Brian D. Shelmadine, Jennifer J. Moreillon, Erika Deike, Jackson O. Griggs, Ronald L. Wilson

**Affiliations:** a Baylor University, Waco, TX, USA; b Texas Lutheran University, Austin, Texas, USA; c Family Medicine, Family Health Center. Waco, Texas, USA; d Central Texas Nephrology Associates, Waco, Texas, USA

**Keywords:** Uric acid, Cholesterol, Chronic kidney disease, LDL, HDL

## Abstract

**Background:**

Few studies have been conducted that compared lipid levels and uric acid in CKD or End-Stage Renal Disease (ESRD) patients with most using animal models. The purpose of the study was to explore effects on lipids while controlling uric acid levels in CKD patients.

**Methods:**

Twenty-four CKD patients (N = 24) volunteered to participate in this study. The study was conducted using a double-blind, randomized, placebo controlled experimental protocol. The experimental group was prescribed 300 mg of allopurinol PO daily by their treating physician and followed prospectively for 8-weeks. The control group consumed a similar pill once a day for 8-weeks.

**Results:**

ANCOVA revealed significant differences in total cholesterol (P = 0.009) and Apo B (P = 0.006) with lower levels in the allopurinol group. A trend emerged with LDL (P = 0.052) with lower levels in the allopurinol group. No significant differences were discovered in triglycerides (P = 0.403), HDL (P = 0.762) and total Cholesterol/HDL Ratio (P = 0.455).

**Conclusions:**

After statistically controlling for compliance and inflammation significant differences between groups were observed for total cholesterol and Apo B. In both instances the allopurinol group had lower concentrations than the placebo group. Similarly, a trend was observed in LDL with the allopurinol group having lower concentrations than the placebo group.

## Introduction

Allopurinol is a xanthine oxidase inhibitor that effectively blocks the formation of uric acid and is used to treat gout. However, high concentrations of uric acid (hyperuricemia; ≥ 7 mg/dL in men and ≥ 6 mg/dL in women) have previously been linked to components of metabolic syndrome [1-4], have been proposed to have a role in developing diabetic nephropathies [5], and have been implicated in the development of hypertension and a decrease in renal function [6].

Lowering uric acid levels using allopurinol has been demonstrated to improve glomerular filtration rate (GFR), blood pressure (BP) and c-reactive protein (CRP) levels in patients with normal renal function [6]. In patients with chronic kidney disease (CKD), lowering uric acid has been reported to significantly improve CRP levels, independently slow the progression of renal disease, and reduce cardiovascular events by 71% [7]. Few studies have been conducted that compared lipid levels and uric acid in CKD or End-Stage Renal Disease (ESRD) patients with most using animal models. Nakagawa et al [8] and Khosla et al [9] reported that decreased uric acid through the use of allopurinol decreased serum lipid levels and triglycerides in rats. Additionally, allopurinol used in a pilot study of ESRD patients significantly improved LDL cholesterol and triglyceride levels [10] although earlier research in past decades did not find allopurinol to be effective at reducing serum levels of cholesterol [11, 12]. Yet, other study authors [13] have reported that uric acid plays a role in dyslipidemia through the suppression of lipid peroxidation.

Complete mechanisms for the effectiveness of allopurinol in improving CRP, BP, GFR, lipid and triglyceride levels remain elusive. However, some proposed mechanisms include suppression of lipid peroxidase [13] and decreases in critical lipase activity [8]. Additionally, allopurinol is known to scavenge free radicals and reduce inflammation [13]. Therefore, the purpose of this study was to explore effects on lipids while controlling uric acid levels by means of allopurinol use in CKD patients.

## Methods

### Patients

Twenty-four CKD patients (N = 24) from a family medicine clinic volunteered to participate in this study after being contacted by their primary care physician. Participants completed a medical history questionnaire and completed a general examination by their treating physician to determine whether they met eligibility criteria. Patients were excluded from the study if they were under 18 years of age, had an active illness requiring hospitalization, had a previous allergic reaction to allopurinol or any of its components, positive blood tests for Hepatitis B and C, dementia, malabsorption syndromes, were pregnant, had a life expectancy of less than three months, malignant hypertension, and/or a history of medication non-compliance. Approval to conduct the study was provided by university and clinic institutional review boards. Patients meeting eligibility requirements signed informed consent statements.

### Experimental design

The study was conducted using a double-blind, randomized, placebo controlled experimental design with 12 patients randomly placed in the experimental group and 12 patients randomly placed in the control group for a sample size of twenty-four. All participants completed the study. The experimental group was prescribed 300 mg of allopurinol PO daily by their treating physician and followed prospectively for 8 weeks. The control group consumed a similar pill once a day for 8 weeks that contained rice flour. Patients were monitored weekly by researchers conducting the study through phone consultation and through regularly scheduled appointments to see their treating physician to measure compliance in both the experimental and control groups for the entire length of the study. Compliance in both groups was monitored through the standard practice of pill counting with patients returning their unused medication/placebo pills at the end of the study with those consuming 80% considered compliant [14, 15]. Additionally, compliance with allopurinol was measured through uric acid levels and participants in the experimental group were considered compliant if their uric acid levels decreased by at least 20% during the study [8]. Ten (83.3%) of participants in the experimental group were considered compliant based on a decrease in uric acid of 20% or greater, while seven (58.3%) were compliant in the control group based on consuming greater than 80% of their pills (placebo). Overall seventeen (70.8%) of participants were compliant. A sample size of greater than 23 was chosen to ensure a power of greater than 80% based on formulas published by Freedman [16] in which the alpha level is .05 and the average rate of change in uric acid values is 20%.

### Data collection

Following a 12-hour fast approximately 50 milliliters of blood was collected for analysis at baseline and 8 weeks. Samples were analyzed for levels of uric acid, albumin, inflammatory markers of CRP, Tumor Necrosis Factor-alpha (TNF-α), interleukin-6 (IL-6), triglycerides, total cholesterol, high-density lipoprotein cholesterol (HDL), low-density lipoprotein cholesterol (LDL), total cholesterol/HDL ratio, and apolipoprotein B (ApoB). Blood samples for inflammatory markers was centrifuged within 10 minutes at 2,400 rpm for 10 minutes and stored at -20 °C. Plasma levels were analyzed for TNF-α, IL-6, and CRP using commercially available assays with an iMark microplate reader (Bio-Rad Laboratories, Inc., Hercules, CA). All assays were performed in duplicate using the manufacturer recommended wavelength against a known standard curve depending on the specifications of the protocol. Standard protocol was used with commercially available kits from Cayman Chemical (Ann Arbor, MI) for each cytokine. Lipid profiles (triglycerides, total cholesterol, HDL, LDL, ApoB, and total cholesterol/HDL), albumin levels, and CRP were independently analyzed by Quest Diagnostics (Dallas, TX). Possible adverse events with allopurinol were tracked through the treating physician with no side effects reported by study participants. Additionally there were no changes in ALT, AST, alkaline phosphatase, albumin, and bilirubin during the study.

### Statistical analysis

Independent t-tests were used to ascertain differences at baseline between groups for uric acid, age, gender, race, albumin, CRP, IL-6, TNF-α, LDL, HDL, triglycerides, total cholesterol, total cholesterol/HDL ratio and ApoB. ANCOVA was used to measures differences at 8 weeks between groups for all lipid variables after controlling for known covariates of albumin, CRP, IL-6, TNF-α, and medication/placebo compliance. ANCOVA was used to control for variables that could have affected the outcome of lipids but were not a variable of interest. An independent sample Kolmogorov-Smirnov test of normality was conducted on uric acid. Pearson correlations were calculated between uric acid and lipid variables of total cholesterol, ApoB and LDL. Statistical analyses were performed using SPSS for Windows version 18.0 (SPSS Inc., Chicago, IL). All data is presented as mean ± standard deviation (SD) with P < 0.05 considered significant.

## Results

Independent t-tests revealed no significant differences at baseline for uric acid (P = 0.307), age (P = 0.435), albumin (P = 0.445), CRP (P = 0.717), IL-6 (P = 0.327), TNF-α (P = 0.703), triglycerides (P = 0.408), total cholesterol (P = 0.540), HDL (P = 0452), LDL (P = 0.707), total cholesterol/HDL ratio (P = 0.741), and ApoB (P = 0.886). Chi Square revealed no significant differences in gender (P = 0.667) and race (P = 0.197) between groups. An independent sample Kolmogorov-Smirnov test of normality for uric acid revealed a normal distribution (P = 0.518). Results are presented in Table 1.

**Table 1:** Demographic and Pretest Values for Treatment and Control Groups

Variable	Allopurinol Group (n = 12)	Placebo Group (n = 12)	P-value
Gender, n (%)			0.667
Females	3 (25.0)	7 (58.3)	
Males	9 (75.0)	5 (41.7)	
Age, y (SD)	59.2 (10.1)	62.4 (9.5)	0.307
Ethnicity, n (%)			0.197
African American	4 (33.3)	8 (66.7)	
Caucasian	4 (33.3)	1 (8.3)	
Hispanic	4 (33.3)	3 (25.0)	
Weight (kg), mean (SD)	86.31 (14.10)	88.35 (31.54)	0.138
Systolic BP, mean (SD)	142.17 (14.30)	139.25 (30.07)	0.764
Diastolic BP, mean (SD)	76.17 (9.97)	73.67 (15.92)	0.649
GFR, mean (SD)	47.30 (15.90)	45.10 (19.80)	0.503
Diabetes, n (%)	8 (66.7)	5 (41.7)	0.154
CRP, mean (SD)	7.73 (2.06)	8.87 (3.18)	0.717
IL-6, mean (SD)	21.76 (14.43)	17.38 (12.46)	0.327
TNF-α, mean (SD)	22.10 (9.91)	23.33 (11.76)	0.703
Albumin, mean (SD)	4.24 (0.24)	4.11 (0.43)	0.445

CRP, uric acid, and albumin are measured in mg/dL; IL-6, IL-1β, and TNF-α are measured in pg/mL.

ANCOVA revealed significant differences in total cholesterol (P = 0.009) and Apo B (P = 0.006) with lower levels in the allopurinol group. A trend emerged with LDL (P = 0.052) with lower levels in the allopurinol group. No significant differences were discovered in triglycerides (P = 0.403), HDL (P = 0.762), total Cholesterol/HDL Ratio (P = 0.455) and uric acid (P = 0.185) between groups. Results are presented in Table 2.

**Table 2:** Pretest (t-Test) and Posttest (ANCOVA) Lipid Values for the Allopurinol Group and the Control Group

Variable*	Allopurinol Group (n = 12)	Placebo Group (n = 12)	P-value
Pre HDL	39.08 (13.48)	35.75 (7.30)	0.452
Post HDL	36.40 (14.72)	38.30 (9.63)	0.762
Pre LDL	97.00 (40.68)	114.92 (34.77)	0.707
Post LDL	96.10 (32.46)	119.56 (42.05)	0.052
Pre Triglycerides	126.83 (42.88)	135.25 (75.27)	0.408
Post Triglycerides	161.20 (89.67)	176.50 (117.96)	0.403
Pre Total Cholesterol	161.42 (38.23)	177.58 (41.13)	0.741
Post Total cholesterol	164.80 (35.97)	188.00 (44.46)	0.009
Pre Total Cholesterol- HDL Ratio	4.55 (1.92)	5.21 (1.88)	0.405
Post Total Cholesterol- HDL Ratio	5.06 (2.15)	5.18 (1.70)	0.455
Pre Apolipoprotein B	78.58 (25.96)	92.25 (22.86)	0.886
Post Apolipoprotein B	79.30 (24.15)	100.30 (25.46)	0.006
Pre Uric Acid	7.73 (2.06)	8.87 (3.18)	0.307
Post Uric Acid	6.50 (1.78)	8.61 (3.30)	0.034
Pre GFR	47.30 (15.90)	45.10 (19.80)	0.407
Post GFR	45.19 (13.56)	41.15 (18.76)	0.578
Pre Creatinine	1.57 (0.61)	2.10 (0.88)	0.307
Post Creatinine	1.62 (.64)	2.11 (0.97)	0.185

HDL, LDL, total cholesterol and apolipoprotein B are measured in mg/dL.

**Figure 1:**
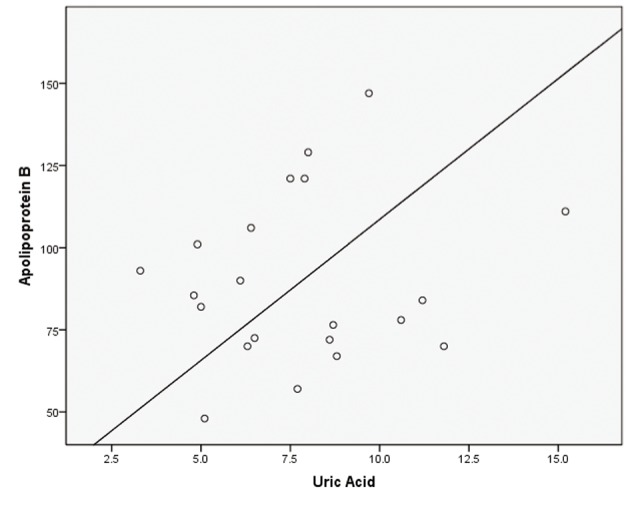
Simple scatterplot for uric acid and apolipoprotein B.

**Figure 2:**
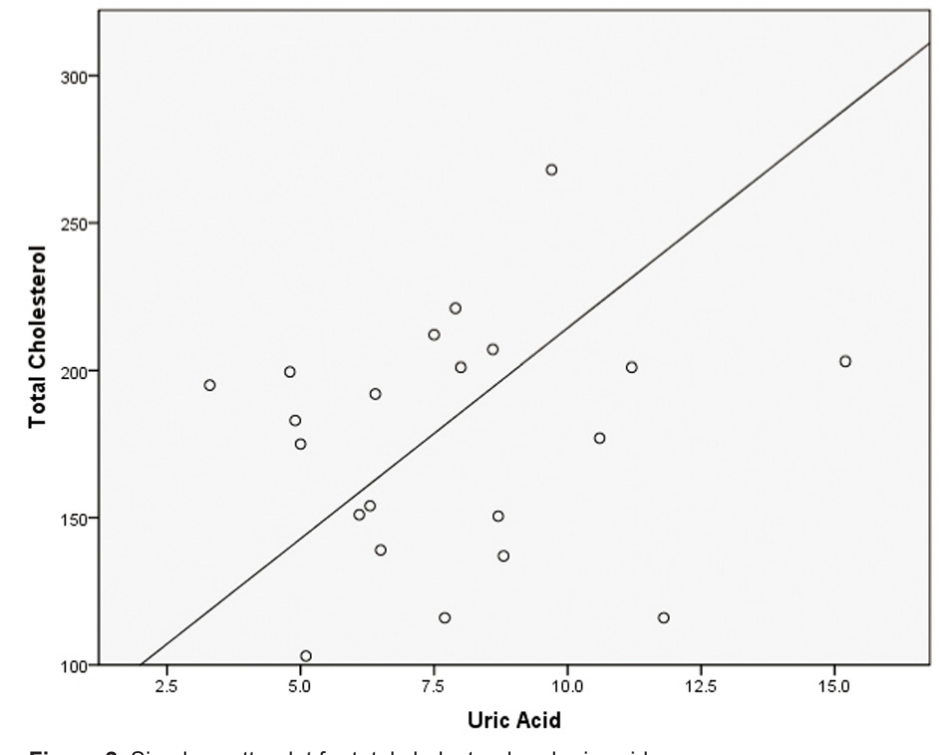
Simple scatterplot for total cholesterol and uric acid.

**Figure 3:**
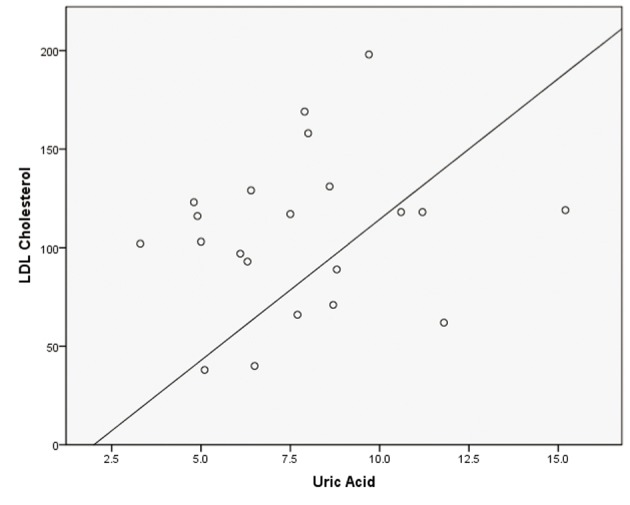
Simple scatterplot for LDL and uric acid.

Pearson correlations were calculated with posttest values revealing week correlations between uric acid and total cholesterol (r^2^ = 0.100), uric acid and ApoB (r^2^ = 0.142), and uric acid and LDL (r^2^ = 0.121) Scatterplots were created using posttest uric acid and posttest lipid values that were discovered to be statistically different with results presented in Figure 1-3.

## Discussion

Hyperuricemia has been linked to an increased risk for cardiovascular disease in patients with normal renal function and an accelerated progression of chronic kidney disease [6, 10]. While there is still discussion as to whether uric acid is a marker of increased risk or whether it serves a causative role [17], recent evidence indicates uric acid may in fact have a causative role [1]. However, most research has focused on mechanisms that may lead to hypertension and/or increased levels of inflammation through lowering uric acid levels [6]. Only a handful have sought to explore the effects of lowering uric acid on lipid levels as studies show elevated lipid levels in hyperuricemic patients with both normal [6] and impaired renal function [10] and obese patients [18] further providing a link to hyperuricemia and metabolic syndrome. Therefore, the purpose of this study was to explore effects on lipids while controlling uric acid levels by means of allopurinol use in CKD patients. After controlling for compliance, albumin, CRP, IL-6, and TNF-α significant differences between groups after eight weeks were observed for total cholesterol and Apo B. In both instances the allopurinol group had lower concentrations than the placebo group. Similarly, a trend was observed in LDL with the allopurinol group having lower concentrations than the placebo group. However, no significant differences were observed between the groups at baseline. These findings support previous research in humans [10] and animal models [8, 9].

The most interesting finding is the reduction ApoB in the experimental group. The Third Report of the National Cholesterol Education Program Expert Panel on Detection, Evaluation, and Treatment of High Blood Cholesterol in Adults [19] suggests that the reduction of LDL is important but must also be accompanied by reductions in LDL particle numbers and smaller LDL particles. ApoB is an indirect measure of LDL particle number and suggests the reduction of uric acid may play a role in helping to produce fewer LDL particle numbers and fewer small and dense atherogenic LDL particles. It should be noted that a trend emerged with LDL that also demonstrated a decrease in LDL cholesterol with a decrease in uric acid. Additionally, triglycerides increased in both groups which is supported by previous research [10] and may be associated with an increase in lipoprotein lipase (LPL) to help control LDL particle number and LDL but may have not been enough to overcome advanced CKD disease and the effects of gout with both disease states normally experiencing elevated triglycerides [10]. LPL is the principle enzyme associated with lipolysis of triglycerides and may be affected by high levels of inflammation in this study population [20]. Though small and dense LDL particles are normally associated with elevated triglycerides, the findings in this study and other study authors suggest the decrease of uric acid may play a role in the enzymatic activity of LPL [10, 12, 17]. Uric acid is major cause of oxidative stress, reduced nitrous oxide release and combined with an increase in activity of LPL may cause higher lipid levels and particle numbers [20]. It should be noted that gout is associated with oxidative stress and LDL plays a significant role in the progression of heart disease in both healthy and gout populations [21]. Krishnan et al [21] reports that uric acid can act as pro-oxidant or an antioxidant, which is dependent upon the state of the oxidized lipoprotein. The authors further suggest that when LDL is mildly oxidized uric acid becomes a pro-oxidant and may be related to lipid hyperoxides. Iso-O et al [22] suggests that oxidative stress can induce the peroxidation of both lipids and lipid hyperoxides which can then produce oxidative stress in the cell. Uric acid when combined with oxidized LDL may cause there to be increases in ApoB and LDL particle numbers and the reason why the use of allopurinol can help decrease some lipoproteins. Yet the finding of both an increase in triglycerides and a decrease in ApoB does not correspond well with previous studies in non-CKD patients [23].

Another important finding of this study was the decrease in total cholesterol in allopurinol group. Though LDL is a strong predictor of heart disease, total cholesterol is also an independent predictor of risk. Previous findings have suggested that hyperuricemia occurs in patients with ESRD and the comorbid condition of gout. High uric acid levels have been associated with insulin resistance, hypertension, endothelial dysfunction, and renal impairment as well as in dyslipidemia, especially in patients with renal impairment [6]. Furthermore hyperuricemia is thought to impair endothelium dependent vasodilatation primarily through lipid oxidation that can cause an increase in total cholesterol [20]. Finally, Ellestad et al [17] suggest that hyperuricemia is a reflection of the degree of xanthine oxidase activation which is derived from oxygen free radical production. This cascade of events might be associated with an increase in total cholesterol suggesting allopurinol as means to control total cholesterol. Our study supports these findings indirectly as total cholesterol values were lower in the group that had lower uric acid levels through means of allopurinol use.

Though the findings of no significant changes in inflammatory markers was not the primary outcome of our study they are not in congruence with previous literature indicating that treatment of hyperuricemia with allopurinol reduces inflammation [24]. Differences in results may be due to the short duration of study as well as the population studied. Additionally, high inflammation levels are associated with a decrease in HDL which may help explain our non-significant findings with HDL [20]. The duration of the current study, eight weeks, may not have provided adequate time to affect inflammation levels. Additionally, CKD patients often have a myriad of co-morbidities and complications, such as diabetes or coronary heart disease that may have hindered the effect of lowering uric acid levels on inflammation.

A few limitations existed for this study and are worthy of note. The sample size was small making inferences more challenging in finding significance and evident trends. Also, the small sample size can lead to Type I errors with an over or underestimation of results. Secondly, some patients presented with Type II diabetes which may have been a confounding variable. By controlling for known confounding variables a clearer picture was presented regarding cholesterol and uric acid. Future studies should include a larger sample size with patients followed for a longer study period. Future studies will help to demonstrate if uric acid is simply a confounding variable or plays a causative role in controlling lipid levels.

### Conclusions

The significant differences in total cholesterol and Apo B as well as the trend in LDL concentrations are novel findings suggesting that compliant patients ingesting 300 mg/day of allopurinol may experience a decrease in lipid levels and reduction in heart disease risk. This novel approach for controlling lipids in CKD may provide another means to reduce heart disease risk in a population that has a rate of dyslipidemia that has been reported to be triple of apparently healthy populations [21].
